# Recent Role of Microorganisms of the *Mollicutes* Class in the Etiology of Bovine Respiratory Disease

**DOI:** 10.3390/pathogens13110951

**Published:** 2024-10-31

**Authors:** Katarzyna Dudek, Robin A. J. Nicholas

**Affiliations:** 1 Department of Cattle and Sheep Diseases, National Veterinary Research Institute, 57 Partyzantów Avenue, 24-100 Pulawy, Poland; 2 The Oaks, Nutshell Lane, Farnham, Surrey GU9 0HG, UK; robin.a.j.nicholas@gmail.com

**Keywords:** *Mollicutes*, bovine respiratory disease, cattle

## Abstract

Bovine respiratory disease (BRD) inflicts significant losses in cattle farming worldwide and is caused by the co-occurrence of various infectious agents which is often compounded by environmental factors. It is well known that microorganisms of the *Mollicutes* class are responsible for respiratory disorders in cattle, including BRD. This review highlights the current role of these microorganisms, in particular *Mycoplasma bovis* and *Mycoplasma dispar*, in the etiology of this disease complex, which has recently shifted toward a primary or predominant cause of the disease.

## 1. Introduction

Bovine respiratory disease (BRD) is responsible for extensive losses in cattle breeding worldwide due to high morbidity and mortality rates [1]. The clinical signs of BRD are non-specific and mainly concern respiratory disorders, which may be accompanied by other symptoms such as otitis media, fever, depression and anorexia [2]. The etiology of BRD is complex and multifactorial and is caused by both infectious and non-infectious factors related to herd management, climatic conditions or animal transport [1]. The infectious factors of BRD include both bacterial and viral agents, where the dominant role has recently been attributed to bacterial agents, which include microorganisms of the *Mollicutes* class, bacteria from the *Pasteurellaceae* family such as *Pasteurella multocida* (*P. multocida*), *Mannheimia haemolytica* (*M. haemolytica*) or *Histophilus somni* (*H. somni*) and others. Additionally, the most frequent viruses linked to BRD are bovine viral diarrhea virus (BVDV), bovine respiratory syncytial virus (BRSV), bovine parainfluenza virus 3 (PI-3V, BPIV3) and bovine coronavirus (BCV, BCoV) [2]. 

Numerous studies indicate that BRD affects various age groups and sectors of cattle. Losses due to BRD in the cattle feedlot sector in the United States have been estimated at more than USD 4 billion annually [3]. This is confirmed by the fact that most injectable antimicrobials are used to treat BRD in the feedlot industry [4].

Microorganisms of the *Mollicutes* class, which includes genera that are proven pathogens of cattle, such as *Mycoplasma* or *Ureaplasma*, are characterized by small genome size and the absence of a cell wall [5]. Many studies have demonstrated that they are associated with respiratory disorders in cattle [5,6] but their role in the etiology and pathogenesis of BRD has been ambiguous for a long time. However, recently, their participation as main causative infectious agents has been confirmed (Table 1). Various clinical specimens and techniques have been used to detect these diagnostically difficult pathogens and these are summarized in Table 2 and Table 3. Recently, the important role of *Mollicutes* bacteria in the etiology of BRD in feedlot cattle has been described [4,7,8,9,10]. In one recent study of feedlot cattle mortalities due to BRD, *Mycoplasma* was one of the most abundant bacterial genera. Detailed analysis showed that the genus *Mycoplasma* was found to be more abundant in all types of samples tested, especially in lungs from the BRD cases compared to the controls, and it is considered the predominant bacterial cause [11].

## 2. *Mycoplasma bovis*

*Mycoplasma bovis*, first isolated in 1961, has long been associated with a range of clinical diseases including mastitis, arthritis, keratoconjunctivitis and calf pneumonia. Because of the numerous viral and bacterial pathogens linked to BRD, its role was overlooked for many years owing to the difficulties in identifying this relatively fastidious organism in diseased tissues. It is now well accepted as one of the primary pathogens of BRD and found wherever cattle are kept, particularly in feedlot and other intensive cattle-rearing systems. Recent studies have confirmed the undisputed role of *M. bovis* in the etiology of BRD. One of these studies covered 156 BRD outbreaks from 120 farms within 30 provinces of Spain [13]. Only diseased animals showing symptoms of BRD were analyzed. The animals came from different age groups, i.e., pre-weaned calves, fattening beef calves, adults and unclassified animals. The presence of nine BRD-associated pathogens, including viruses (BCV, PI-3, BRSV, BVDV and BHV-1) and bacteria (*M. bovis*, *P. multocida*, *M. haemolytica* and *H. somni*), was determined in the tested samples using individual q-PCRs. In this study, *M. bovis* was the second most common pathogen after *P. multocida* identified from the BRD outbreaks. *M. bovis* was detected in 77% of 121 outbreaks with a confidence interval of 95% ranging between 70.2% and 83.6%. Based on the frequency of detection of the tested respiratory pathogens, the outbreaks were divided into two clusters using a hierarchical cluster analysis. In cluster 1, *M. bovis* and identified viruses were detected more frequently than in the total number of outbreaks. In cluster 2, the percentage of *M. bovis* and viruses detected was significantly lower. It is worth emphasizing that in cluster 2, where the presence of identified viruses was much lower than in cluster 1, the share of *M. bovis* was still significant (72.6% of the 106 outbreaks). Further analysis, including animal category and seasonality, showed that significantly more BRD outbreaks occurred in pre-weaned calves during winter (between December and March) in cluster 1 compared to cluster 2, where the lack of seasonality and the dominance of outbreaks in older animals (fattening beef calves) were observed. Overall, *M. bovis* appeared to be the primary cause of BRD in cluster 2 outbreaks, although the frequency of its detection in cluster 1 outbreaks was also significant; however, *M. bovis* always co-infected with the viral agent [13].

A subsequent study confirmed the significant role of *M. bovis* alongside viruses associated with BRD in the etiology of the disease [14]. This study examined nasal swabs from 89 calves with clinical signs of BRD from 28 Japanese farms. All calves were tested for the presence of 12 BRD-associated pathogens, including eight viruses—BVDV, BCoV, bovine torovirus (BToV), bovine adenovirus (BAdV), BRSV, BPIV3, bovine influenza D virus (BIDV) and bovine herpes virus 1 (BHV1) as well as four bacteria: *M. bovis*, *M. haemolytica*, *P. multocida* and *H. somni*, using multiplex real-time RT-PCR (multiplex RT-qPCR). In the calves with clinical signs consistent with BRD, *M. bovis* was detected in 23.6% of 89 samples and was most often detected in co-infection with other microorganisms, both viral and bacterial. Single detection of *M. bovis* was observed in two diseased calves [14].

A significant role for *M. bovis* in co-infection with *M. haemolytica* in the acute stage of BRD was demonstrated in feedlot cattle in the US [8]. Apart from *M. bovis*, the presence of other BRD-associated pathogens, such as BRSV, *H. somni*, *M. haemolytica* and *P. multocida*, was determined in the tested samples. This study showed a subsequent increase in the prevalence of *M. haemolytica* in the upper respiratory tract of feedlot cattle following infection by *M. bovis*. An increased prevalence of *M. bovis* was observed in the initial period (during the first two weeks) after the arrival of cattle to the feedlot; hence in this study *M. bovis* had not only a direct role in the etiology of acute BRD, but also an impact on the composition of the respiratory microbiome [8].

An analysis of the nasal microbiome in 58 BRD-affected steers kept in one feedlot in the US showed a significant role for *M. bovis* in disease development [12]. In this study, an increase in the relative abundance of *Mycoplasma* spp. in the BRD cases was observed. *Mycoplasma* spp. was the third most relative abundant genus in the nasal microbiome of cattle displaying BRD, following the genera *Mannheimia* and *Moraxella*. However, the genera *Moraxella* and *Mannheimia* were also among the four most relative abundant genera in healthy steers, in contrast to the genus *Mycoplasma*. Detailed analysis demonstrated a higher prevalence and abundance of *M. bovis* in the nasal cavity of BRD-affected animals compared to healthy ones. Similar results were observed for *M. haemolytica*, which may indicate the co-occurrence of the bacteria in the BRD cases. Additionally, the association between *M. bovis* and *Corynebacterium* was shown in the BRD steers with higher co-occurrence probability than in healthy animals [12].

The important role of *M. bovis* in the etiology of BRD in feedlot cattle in Canada was confirmed in a recent study [9]. Regardless of the type of samples analyzed (nasopharyngeal or trans-tracheal samples), a significantly higher frequency of *M. bovis* was detected in cattle with BRD compared to healthy animals, with tracheal samples showing the highest prevalence. Detailed analysis including other bacteria responsible for BRD, such as *P. multocida*, *M. haemolytica* and *H. somni*, showed that *M. bovis* was the second most common pathogen after *P. multocida* in the analyzed BRD cases [9,20]. 

The role of *M. bovis* in the etiology of BRD in imported bulls was demonstrated in an observational study involving 264 animals intended for fattening in Italy [10]. On arrival, almost half of the animals examined showed clinical respiratory symptoms including nasal discharge, and, to a lesser extent, cough and ocular discharge. *M. bovis* was detected in almost 80% of 88 pooled nasal swabs tested by RT-PCR and in over 95% of 44 pools analyzed by culturing, on days 2 and 15, respectively, after the arrival of the bulls at the beef fattening unit [10].

In cases of, among others, untreated chronic pneumonia in feedlot cattle, a significant role of *M. bovis* has been demonstrated [4]. The examined steers were segregated from cattle kept in one Canadian feedlot. In these cases, *M. bovis* was the second most frequently detected BRD pathogen after *P. multocida*, being detected in 48% of the samples tested. *M. haemolytica* was also identified, although at a low frequency. This study showed that co-isolation occurred in 40% of the cases studied. Detailed analysis using metagenomic sequencing confirmed the significant role of *M. bovis* as one of the most abundant BRD pathogens detected. This method demonstrated the presence of *M. bovis* in 52% of the samples tested, and it was more sensitive than culture. However, the analysis of culture and sequencing showed inter-method concordance in only eight cases. In four other cases, the presence of *M. bovis* was detected only by culture, while for five other samples the mycoplasma was detected only by metagenomic sequencing. These results highlight the importance of using different methods in parallel, especially for the detection of this mycoplasma. Additionally, sequencing was able to detect other bacteria, some of them previously undetected by culturing in the tested samples, including *M. dispar*, other *Mycoplasma* spp., *H. somni*, other *Mannheimia* spp., *Moraxella bovis*, *Moraxella bovoculi*, *Bibersteinia trehalosi* and others. In each of the tested samples, the co-occurrence of two or more detected pathogens was shown [4].

A further study has confirmed the important role of *Mollicutes* bacteria, especially *M. bovis*, in the etiology of BRD in feedlot cattle [7]. The study was conducted on a large group of imported bulls intended for fattening on 13 different Italian farms. In almost 70% of tested nasal samples, microorganisms of the *Mollicutes* class were isolated, including *M. bovirhinis*, *M. bovis*, *M. dispar*, *M. arginini*, *M. alkalescens*, *M. ovipneumoniae*, *M. fermentans*, *Ureaplasma* spp. and *Acholeplasma laidlawii*. Most of them were found in mixed cultures. This study showed a generally increasing frequency of *Mollicutes* isolation over time, although it varied between the farms sampled. From the pool of all the analyzed nasal samples, *M. bovis* was the second most frequently detected pathogen of the *Mollicutes* class, in over 19% of samples, including almost 7% of pure cultures. This percentage increased to almost 40% after the analysis of the tested samples using the *M. bovis*-specific PCR. This study showed that *M. bovis* prevalence in cattle was time-dependent and varied largely between farms, regardless of the method used for its detection. A general increase in the percentage of both isolation and PCR-positive frequency was observed at day 15 post-arrival of the animals, in contrast to the 60th day of sampling, when this frequency decreased in most cases. It is worth emphasizing, however, that unlike other *Mycoplasma* such as *M. dispar*, *M. bovis*’s prevalence in the bulls on their arrival was relatively low in most of the farms sampled. Its increase after two weeks is almost certainly due to the ideal environment provided by the feedlot system, enabling the rapid spread of this microorganism between animals on the farm, especially in the first weeks of the fattening period [7]. 

Other studies have demonstrated a significant role of *M. bovis* in the development of BRD in lactating dairy cows in Brazil [15]. This is important because infections with *M. bovis* in cows are usually associated with mastitis rather than respiratory disease. In this study, nasal swabs were tested for nine BRD-associated pathogens: *M. bovis*, *H. somni*, *P. multocida*, *M. haemolytica*, BVDV, BRSV, BPIV3, BCoV and bovine alpha herpesvirus 1 (BoAHV-1). The results showed the presence of *M. bovis* DNA alone or in co-infection in over 50% of acute respiratory cases in two high-yielding dairy herds (40% in herd 1 and almost 88% in herd 2). Concomitant infections involved only bacterial agents with no viruses detected in the nasal swab samples. This could have been influenced by the vaccination program practiced in both herds including the tested viruses, except BCoV. In herd 1, single infections with *M. bovis* or *H. somni*, as well as dual infections of *M. bovis* and *H. somni*, were observed. In the second herd, one single case of infection with *M. bovis* was found, along with co-infections with two bacteria including *P. multocida*, and, less frequently, *H. somni*. The role of *M. bovis* in co-infections in both herds was significant and amounted to 35% and 75% in herds 1 and 2, respectively. Such a clear contribution of *M. bovis* to BRD cases indicates the direct role of this pathogen not only in the chronic but also in the acute stages of this disease [15].

However, most studies used nasal or nasopharyngeal swabs as a specimen, which, in the case of these bacteria that have the ability to colonize the upper respiratory tract, often without causing clinical symptoms of the disease, is not always clear evidence of their role in the development of BRD. Immunocytochemical staining of lungs infected with *Mycoplasma*, on the other hand, may provide the best approach for correlating the location of the pathogen with the caused lung damage [6,21,22].

## 3. *Mycoplasma dispar*

While *M. dispar* was shown to cause a mild pneumonia following experimental infection, it has been difficult to definitively associate it with BRD in the field [6]. Thirty years ago, ter Laak et al. [23] provided some supporting evidence when they detected *M. dispar* in 92% of pneumonic calf lungs but in only 40% of healthy lungs. More recently, a study conducted in Brazil on calves with clinical signs of BRD supported its role in the development of this disease [16]. Samples were tested for the presence of *Mollicutes* including selected *Mycoplasma* species such as *M. bovis*, *M. dispar* and *Mycoplasma mycoides* subsp. *mycoides* (*Mmm*). Mollicute genetic material was detected in the BRD calves at a significantly higher frequency than in healthy animals. Detailed species analysis detected *M. dispar* DNA in more than 60% of BRD cases, which was significantly higher than in the healthy calves. A significant association between the occurrence of clinical symptoms of BRD and the presence of *M. dispar* DNA was found in the case of tachypnea and mixed dyspnea. In comparison, *M. bovis* was identified in only one calf that showed clinical signs of BRD, while the presence of *Mmm* DNA was not detected in any of the animals tested [16].

The prevalence of *M. dispar* in the tested samples was also demonstrated in a study involving imported bulls stabled at Italian fattening farms, where the share of *M. bovis* was examined [7]. In this study, *M. dispar* was the third most common *Mycoplasma* species detected, accounting for just over 12% of all analyzed nasal swabs. Pure cultures of this bacterium were obtained in over 7%. In contrast to *M. bovis*, the *M. dispar* frequency was not dependent on time, but it varied similarly between the tested farms, although to a lesser extent [7].

In a study evaluating the role of BRD pathogens in nonresponsive pneumonia or lameness cases in feedlot cattle in Canada, the presence of *M. dispar* was not demonstrated by culture, probably due to its fastidiousness in culture; but culture-independent techniques like metagenomic sequencing detected this mycoplasma in all samples tested [4,6]. It was shown to co-occur with other *Mycoplasma* or another BRD-associated microorganisms. This study also showed the frequent co-occurrence of *M. dispar* with microorganisms of the *Pasteurellaceae* family, especially *P. multocida*, which confirms the possible synergistic effect of these BRD-associated bacteria [4].

## 4. *Mycoplasma bovirhinis*

Since first reported in 1967, *M. bovirhinis* has been detected in the upper and lower respiratory tracts of both healthy and diseased cattle throughout the world [6]. It has never been thought to be a primary pathogen but may exacerbate existing disease conditions caused by other pathogens, including *M. bovis* and *M. dispar*. Lately, one study assessing the role of *Mycoplasma* infections in the etiology of BRD was conducted in Brazil on 103 suckling calves with clinical symptoms of the disease [17]. In this study, a similar number of clinically healthy calves were also tested. Nasal swabs collected from all the calves were examined for pathogens associated with BRD, such as bacteria of the *Mollicutes* class, *Pasterellaceae* family (*P. multocida*, *M. haemolytica* and *H. somni*) and selected viruses: BVDV, BRSV, BPIV3, BoAHV1, BCoV and ovine gammaherpesvirus 2 (OvGHV2). Although *M. bovirhinis* was the most frequently detected BRD-associated pathogen among those examined in the clinical cases, its real role in the etiology of BRD in this study was not proven due to its presence in a comparable percentage in the asymptomatic calves. However, *M. bovirhinis* was detected in all examined cattle farms. It is worth emphasizing that most cases of singular infections in the diseased calves were caused by *M. bovirhinis*. However, it was similar to the case of asymptomatic calves in which *M. bovirhinis* was detected as a single infectious agent most often, and in total these cases were more numerous. However, the share of dual infections with *M. bovirhinis* and OvGHV2 or BCoV was higher in the symptomatic calves compared to the asymptomatic ones. It was also similar in the case of quadruple infections due to *M. bovirhinis*, OvGHV2, BCoV and *P. multocida*. The specific genes of viruses considered to be associated with BRD, such as BCoV and OvGHV2, were identified in a high percentage of cases, but at a lower frequency than *M. bovirhinis*. However, their percentage share in both the diseased and clinically healthy animals was also similar to those in the case of *M. bovirhinis*, especially for BCoV. Unexpectedly, the genetic material of other *Mollicutes*, including *M. bovis* and bovine viruses such as BVDV, BRSV, BPIV3 and BoAHV1, was not identified in any of the tested samples [17].

In the study where the prevalence of both *M. bovis* and *M. dispar* was observed in the cases of BRD in imported bulls in Northern Italy, the presence of *M. bovirhinis* was also identified in the tested nasal swabs [7]. From the pool of all the analyzed samples, *M. bovirhinis* was the most frequently detected *Mollicutes* bacteria (in almost 40% of samples), including over 18% of pure cultures. Similarly to *M. bovis*, the frequency of *M. bovirhinis* was characterized by high variability within the studied farms. In contrast to *M. bovis*, a general increase in the percentage of *M. bovirhinis* frequency was observed at day 60 post-arrival. Additional analysis showed that the *M. bovirhinis* prevalence was season-dependent with a higher frequency of this mycoplasma in the warm season [7].

## 5. *M. bovigenitalium*

*Mycoplasma bovigenitalium*, first characterized in 1955, is more often found in the reproductive tract of cattle and buffaloes where it may be associated with endometritis, reduced fertility and granular vulvitis [6]. However, there have also been reports of isolation from the lungs of pneumonic but not healthy calves [23]. Unfortunately, until recently there has been little research into the role of *M. bovigenitalium* in BRD. However, a study carried out in Egypt on sixty calves with respiratory symptoms showed a possible role of *M. bovigenitalium* in the development of BRD [18]. The samples were tested for the most common respiratory pathogens of bacterial origin in the study area, such as *M. bovis*, *M. bovigenitalium*, *P. multocida* and *Staphylococcus aureus* (*S. aureus*). Of the pool of sixty samples, six were positive for the tested bacteria, including five with confirmed presence of at least one *Mycoplasma* agent. In all of these cases, mixed infections were encountered, which was the result of dual or triple infections. The most frequently identified bacteria from the positive cases was *M. bovis* (over 8% of all samples tested), while the remaining bacterial agents, including *M. bovigenitalium*, were detected in 5% of all cases studied. *M. bovigenitalium* was identified in co-infection with *M. bovis* alone or together with *S. aureus* [18]. 

## 6. *Ureaplasma diversum*

Another microorganism of the *Mollicutes* class more often associated with reproductive disease, *U. diversum*, has also been linked with BRD. Although only causing a subclinical respiratory disease following experimental infection in gnotobiotic calves, typical “cuffing” lesions were seen surrounding air passages and blood vessels [24]. Recent studies conducted on Australian feedlot cattle have shown a significant role for *U. diversum* in cases of BRD as an opportunistic pathogen for cattle undergoing targeted treatment [19]. The study included diseased animals classified as BRD, as well as non-BRD cases and apparently healthy cattle in the feeding period. Nasal swabs were tested for the urease subunit-γ gene of *U. diversum* and other pathogens associated with BRD, such as *M. bovis*, *P. multocida*, *M. haemolytica*, *H. somni*, *T. pyogenes* and BoAHV1. During the 14-day feeding period, a slight increase in the prevalence of *U. diversum* in nasal swabs was observed in healthy animals. However, compared to the diseased animals, it was more than six times lower. Additionally, in the diseased animals, the prevalence of *U. diversum* was not dependent on the reason for treatment (BRD or non-BRD cases). In the BRD cases, the most common pathogen associated with *U. diversum*, in as many as 90% of cases, was *M. bovis*. These bacteria were detected simultaneously with one or more of the pathogens tested. In one case, the coexistence of *U. diversum* and *P. multocida* was detected [19].

## 7. Conclusions

This review strongly confirms the pathogenic role of some microorganisms of the *Mollicutes* class, especially *M. bovis* and *M. dispar*, in the etiology of BRD. Further evidence could be provided by screening the lungs for the presence of *Mycoplasma* agents, which would be evidence of colonization of the lower respiratory tract. However, most studies used nasal or nasopharyngeal swabs as a specimen, which, in the case of bacteria that have the ability to colonize the upper respiratory tract, often without causing clinical symptoms of the disease, is not always clear evidence of their role in the development of BRD. Immunocytochemical staining of lungs infected with *Mycoplasma*, on the other hand, may provide the best approach for correlating the location of the pathogen with the caused lung damage [6,21,22]. The combination of a number of methods and clinical specimens, mainly in the case of intravital diagnostics, may be the key to a reliable assessment of the role of microorganisms of the *Mollicutes* class in the etiology of the disease.

## Figures and Tables

**Table 1:** *Mollicutes* microorganisms responsible for BRD.

Pathogen	References
*Mycoplasma* spp.	[11]
*Mycoplasma bovis* (*M. bovis*)	[4,7,8,9,10,12,13,14,15]
*Mycoplasma dispar* (*M. dispar*)	[4,7,16]
*Mycoplasma bovirhinis * (*M. bovirhinis*)	[7,17]
*Mycoplasma bovigenitalium * (*M. bovigenitalium*)	[18]
*Ureaplasma diversum * (*U. diversum*)	[19]

**Table 2:** Clinical specimens for detection of *Mollicutes* microorganisms.

Specimen	Pathogen Isolated	Animal Category or Sector of Cattle	References
Nasal swab	*M. bovis*	Dairy cattle	[15]
		Beef cattle	[10]
		Feedlot cattle	[7,8,12]
		Undefined	[14]
	*M. bovirhinis*	Dairy cattle	[17]
		Feedlot cattle	[7]
	*U. diversum*	Feedlot cattle	[19]
	*M. dispar*	Feedlot cattle	[7]
Nasopharyngeal swab	*M. bovis*	Beef cattle	[13]
		Feedlot cattle	[4,9]
		Undefined	[18]
	*M. dispar*	Feedlot cattle	[4]
	*M. bovigenitalium*	Undefined	[18]
	*Mycoplasma* spp.	Beef cattle	[11]
Tracheal scrape	*M. bovis*	Beef cattle	[13]
Trans-tracheal aspiration	*M. bovis*	Feedlot cattle	[9]
Tracheal wash	*M. bovis*	Dairy cattle	[16]
	*M. dispar*		
Transected trachea	*Mycoplasma* spp.	Beef cattle	[11]
Bronchoalveolar lavage	*M. bovis*	Beef cattle	[13]
Lung tissue	*M. bovis*	Beef cattle	[13]
	*Mycoplasma* spp.	Beef cattle	[11]
Synovial tissue	*Mycoplasma* spp.	Beef cattle	[11]

**Table 3:** Methods used for detection of *Mollicutes* microorganisms.

Method	Pathogen Isolated	Animal Category or Sector of Cattle	References
PCR	*M. bovis*	Dairy cattle	[16]
		Undefined	[18]
	*M. dispar*	Dairy cattle	[16]
	*M. bovigenitalium*	Undefined	[18]
Arbitrarily primed polymerase chain reaction (AP-PCR)	*M. bovis*	Feedlot cattle	[7]
Quantitative PCR (q-PCR), real-time PCR	*M. bovis*	Beef cattle	[13]
		Feedlot cattle	[9,12]
Multiplex qPCR with Ta-Man chemistry	*M. bovis*	Feedlot cattle	[8]
Multiplex Real-Time RT-PCR (Multiplex RT-qPCR)	*M. bovis*	Undefined	[14]
Qualitative RT-PCR	*M. bovis*	Beef cattle	[10]
Nested-PCR (nPCR)	*M. bovirhinis*	Dairy cattle	[17]
	*M. bovis*	Dairy cattle	[15]
*de novo* PCR	*U. diversum*	Feedlot cattle	[19]
PCR/Denaturing gradient gel electrophoresis (PCR/DGGE)	*M. bovis*	Beef cattle	[10]
		Feedlot cattle	[7]
	*M. dispar*		
	*M. bovirhinis*		
MALDI-TOF MS	*M. bovis*	Feedlot cattle	[4]
Metagenomic sequencing	*M. bovis*	Feedlot cattle	[4]
	*M. dispar*		
	*Mycoplasma* spp.		
*16S rRNA* gene sequencing	*Mycoplasma* spp.	Beef cattle	[11]
	*M. bovigenitalium*	Undefined	[18]
	*M. bovis*	Feedlot cattle	[12]
*Mb-mp 81* gene sequencing	*M. bovis*	Undefined	[18]
16S-23S ribosomal DNA intergenic region sequencing	*M. bovis*	Dairy cattle	[15]
Direct sequencing	*M. bovirhinis*	Dairy cattle	[17]
Biochemical tests	*M. bovis*	Undefined	[18]
	*M. bovigenitalium*		

## Data Availability

Not applicable.
